# Dissecting the Natural Patterns of Progression and Senescence in Pediatric Low-Grade Glioma: From Cellular Mechanisms to Clinical Implications

**DOI:** 10.3390/cells13141215

**Published:** 2024-07-19

**Authors:** David Gorodezki, Martin U. Schuhmann, Martin Ebinger, Jens Schittenhelm

**Affiliations:** 1 Department of Hematology and Oncology, University Children’s Hospital Tübingen, 72076 Tübingen, Germany; martin.ebinger@med.uni-tuebingen.de; 2 Section of Pediatric Neurosurgery, Department of Neurosurgery, University Hospital Tübingen, 72076 Tübingen, Germany; 3 Department of Neuropathology, Institute of Pathology, University Hospital Tübingen, 72076 Tübingen, Germany

**Keywords:** pediatric low-grade glioma, pilocytic astrocytoma, RAS/MAPK pathway, oncogene-induced senescence, tumor microenvironment

## Abstract

Pediatric low-grade gliomas (PLGGs) comprise a heterogeneous set of low-grade glial and glioneuronal tumors, collectively representing the most frequent CNS tumors of childhood and adolescence. Despite excellent overall survival rates, the chronic nature of the disease bears a high risk of long-term disease- and therapy-related morbidity in affected patients. Recent in-depth molecular profiling and studies of the genetic landscape of PLGGs led to the discovery of the paramount role of frequent upregulation of RAS/MAPK and mTOR signaling in tumorigenesis and progression of these tumors. Beyond, the subsequent unveiling of RAS/MAPK-driven oncogene-induced senescence in these tumors may shape the understanding of the molecular mechanisms determining the versatile progression patterns of PLGGs, potentially providing a promising target for novel therapies. Recent in vitro and in vivo studies moreover indicate a strong dependence of PLGG formation and growth on the tumor microenvironment. In this work, we provide an overview of the current understanding of the multilayered cellular mechanisms and clinical factors determining the natural progression patterns and the characteristic biological behavior of these tumors, aiming to provide a foundation for advanced stratification for the management of these tumors within a multimodal treatment approach.

## 1. Pediatric Low-Grade Glioma: Epidemiology, Classification and Contemporary Treatment Patterns

Pediatric low-grade gliomas (PLGGs) comprise several brain tumor entities of glial and glioneuronal histology assigned to WHO CNS grade 1 and 2 [1,2,3]. These tumors collectively represent the most common CNS tumors of the pediatric population, comprising approximately 30% to 40% of newly diagnosed brain tumor cases in children and adolescents. In Western populations, the cross-entity incidence rate is currently estimated at 2–3 per 100,000 children [4,5]. Occurring in all age groups from infancy to adolescence, PLGGs show a peak incidence in children 5 to 9 years of age [6]. In the current WHO classification of central nervous system tumors published in 2021, PLGGs are categorized into six categories under the umbrella term “glioma, glioneuronal and neuronal tumors” [1,2,3]. Common histological entities include pilocytic astrocytoma and pleomorphic xanthoastrocytoma, which are characterized as circumscribed astrocytic gliomas, alongside several entities categorized as pediatric-type diffuse low-grade gliomas, including the less common MAPK pathway-altered diffuse low-grade glioma or MYB-/MYBL1-altered diffuse astrocytoma [1,2,3]. Among glioneural and neuronal tumors, gangliogliomas and dysembryoplastic neuroepithelial tumors (DNETs) are also considered PLGGs [1,2,3]. PLGGs occur at all sites within the central nervous system, while predominant regions include the cerebellum and the supratentorial midline structures, and entity-specific distribution of predominant tumor locations is observed [6].

A key characteristic of PLGGs is their frequent association with tumor predisposition syndromes including neurofibromatosis type 1 (NF1) and tuberous sclerosis complex (TSC) [7]. In particular, tumors of the visual pathway are associated with NF1 and occur in approximately 20% of these patients within the first decade of life, while almost exclusively pilocytic astrocytomas are found [8,9,10]. Conversely, NF1 can be detected in about 40% of all patients with OPG [10]. Although these patients, specifically in the case of tumors involving the chiasmatic/hypothalamic region, showed superior progression-free survival rates compared to their non-NF1 counterparts, they often present a significant challenge for their caregivers due to increased toxicity to most conventional treatments [11,12].

Despite promising recent advances in the implementation of molecular targeted therapies, surgery remains the mainstay of therapy of PLGGs. The progression-free survival (PFS) is substantially determined by the extent of surgical resection [11,13,14,15,16,17]. In many cases, however, resectability can be severely compromised by surrounding highly eloquent brain tissue, and extensive resection may cause substantial morbidity. Recent population-based cohort studies consistently report high rates of incomplete resection (IR) ranging between 65% and 73%, despite recent advancements in neurosurgical technology [11,14,17,18]. In cases where resection is not feasible, surgical biopsy is recommended, whereas in patients with confirmed NF1 and characteristic MRI findings of an optic pathway glioma, this is not considered obligatory [19].

In the case of limited resectability and progressive disease, chemotherapy has been considered the treatment of choice in recent years. Approximately four decades ago, initial chemotherapy protocols for pediatric low-grade gliomas (PLGGs) were introduced and assessed, with the purpose of postponing radiotherapy and offering an effective substitute treatment for NF1 patients, who inherently face a notably elevated risk of secondary malignancies following radiation therapy [20]. Currently applied chemotherapy regimens include either carboplatin and vincristine, vinblastine monotherapy, or a combination of thioguanine, procarbazine, CCNU, and vincristine [21,22,23,24]. Further second-line protocols include irinotecan and bevacizumab [25]. Notably, however, 5-year PFS rates of established first-line chemotherapy protocols of approximately 50% have been reported, and response rates of subsequent second-line treatments in most cases significantly decrease, causing significant long-term morbidity in affected patients [22,23,24,26].

Given its efficacy in tumor control, radiation therapy has been the primary choice for salvage treatment in PLGGs in previous decades [20]. Considering the substantial age-related long-term sequelae such as cognitive decline, endocrine disorders, and secondary malignancies, conventional photon radiation should currently be reserved for a certain subset of older non-NF1 patients following careful consideration [20,27,28]. This shift has prompted the advancement of alternative radiation applications, including proton beam therapy and stereotactic radiation, as these methods aim to enhance local tumor control while reducing the risk of long-term side effects [20,29].

In recent years, in-depth molecular profiling and incremental decoding of the molecular mechanisms of tumorigenesis in PLGGs has led to clinical evaluation of the efficacy of molecular therapies targeting the RAS/MAPK and mTOR pathways of these tumors [21,30]. In previous phase I/II studies, several agents, including MEK inhibitors (selumetinib, trametinib, and binimetinib), a pan-RAF inhibitor (tovorafenib), first-generation BRAF inhibitors (vemurafenib and dabrafenib), an mTOR inhibitor (everolimus), and an FGFR inhibitor (erdafitinib), have shown promising results, leading to the implementation of randomized controlled studies comparing several agents, including selumetinib, trametinib, and tovorafenib, to the first-line chemotherapy regimen in newly diagnosed, previously untreated PLGGs [31,32,33,34,35,36,37]. It was recently shown that a combination therapy of trametinib and dabrafenib achieved significantly higher response rates compared to carboplatin and vincristine in PLGGs bearing a BRAF V600E mutation and therefore is currently being considered the first-line treatment in these patients [38]. Crucial questions, however, which include the optimal duration of treatment, have not yet been addressed in studies and are the subject of current debate. In a significant subset of patients, rapid rebound growth after discontinuation of therapy has been observed, while potential risk factors have not yet been identified [39]. Remarkably, renewed response to subsequent treatment periods indicates an insignificant role of acquired resistance in these therapies. Significant data regarding potential long-term toxicities, especially in the pediatric demographic, are as yet missing [30]. Apart from MEK inhibitors, other molecular therapies targeting the RAS/MAPK pathway are currently not used in NF1 patients due to concerns involving therapy-related paradoxical ERK activation due to RAF homodimerization, which has previously been observed in vivo and in vitro [40,41].

Recurrent progressions and the necessity for multiple different lines of therapies in many cases emphasize the chronic nature of this disease, which bears a high risk of long-term disease- and therapy-related morbidity in affected patients.

## 2. Multi-Layered Clinical Factors Determine the Natural Progression Patterns of PLGGs

**Figure 1:**
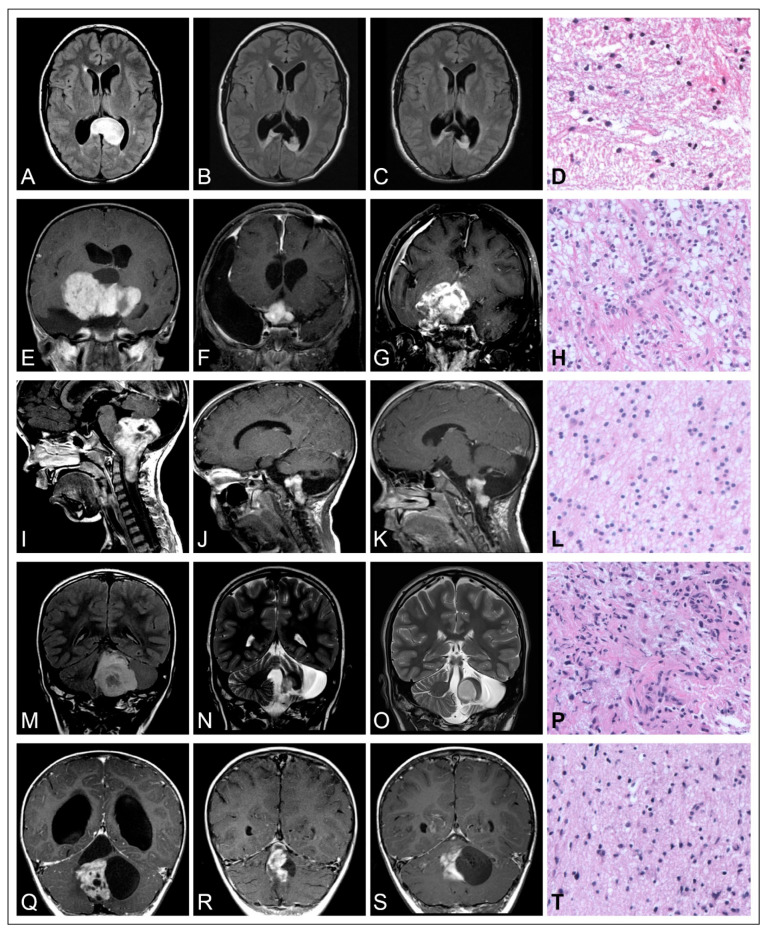
Illustration of the versatile postoperative progression patterns of pediatric low-grade gliomas after incomplete resection (IR). (**A**–**D**) Diffuse astrocytoma WHO grade 2 of the posterior corpus callosum in a 15-year-old girl. (**A**) Preoperative MRI image, (**B**) postoperative MRI image after IR, (**C**) follow-up MRI image 9 years after IR, showing stable tumor remnants. (**D**) H&E stain of the tumor, 200× magnification. (**E**–**H**) Pilocytic astrocytoma (PA) WHO grade 1 of the chiasm and hypothalamus in a 7-year-old girl. (**E**) Preoperative image, (**F**) postoperative image after IR, (**G**) follow-up MRI image 7 years after IR, showing tumor progression under adjuvant treatment. (**H**) H&E stain of the tumor, 200× magnification. (**E**–**H**) Pilocytic astrocytoma (PA) WHO grade 1 of the chiasm and hypothalamus in a 3-year-old girl. (**E**) Preoperative MRI image, (**F**) postoperative MRI image after IR, (**G**) follow-up MRI image 7 years after IR, showing tumor progression under adjuvant treatment. (**H**) H&E stain of the tumor, 200× magnification. (**I**–**L**) Pilocytic astrocytoma (PA) WHO grade 1 of the medulla oblongata in a 4-year-old boy. (**I**) Preoperative MRI image, (**J**) postoperative MRI image after IR, (**K**) follow-up MRI image 2 years after IR, showing stable tumor remnants. (**L**) H&E stain of the hypocellular tumor showing a predominantly fibrillary matrix, 200× magnification. (**M**–**P**) Pilocytic astrocytoma (PA) WHO grade 1 of the cerebellum in a 7-year-old boy. (**I**) Preoperative MRI image, (**O**) postoperative MRI image after IR, (**N**) follow-up MRI image 6 years after IR showing tumor progression. (**P**) H&E stain of the tumor, 200× magnification. (**Q**–**T**) Pilocytic astrocytoma (PA) WHO grade 1 of the cerebellum in a 2-year-old boy. (**Q**) Preoperative MRI image, (**R**) postoperative MRI image after IR, (**S**) follow-up MRI image 4 years after IR, showing stable tumor remnants. (**T**) H&E stain of the solid tumor portions resembling a diffuse growth pattern, 200× magnification.

The clinical course of pediatric low-grade gliomas is commonly characterized by an indolent growth behavior. In a previously reported cohort, it was shown that almost half of patients diagnosed with PLGG exhibited symptoms related to their disease more than six months prior to diagnosis. Again, this emphasizes the indolent growth patterns of these tumors [14]. After incomplete resection, growth deceleration and senescence are frequently observed, as long-term progression-free survival (PFS) rates of approximately 50% were correspondingly reported from previous population-based cohort studies [11,14,42]. Both spontaneous regression as well as regrowth of senescent tumors up to 12 years after initial diagnosis and malignant transformation to secondary high-grade lesions have occasionally been reported [7,14,43,44,45,46,47,48,49,50]. The versatile postoperative progression patterns of pediatric low-grade gliomas after incomplete resection (IR) is illustrated in Figure 1 based on five cases of distinct histological entities and tumor locations.

While the growth behavior and progression patterns of PLGGs are repeatedly described as mostly unpredictable at first glance, the results of a multi-state analysis dissecting the natural course of the disease indicate that future progressiveness may be predicted by the tumor growth behavior during the first two years after initial diagnosis [51]. The comprehensive analysis of more than 1500 patients during multiple disease states identified various levels of disease progressiveness, which were determined by age, tumor location, and histologic grade [51]. Previous population-based cohort studies have identified several risk factors potentially determining progression patterns of these tumors, mainly including the extent of surgical resection, tumor location, age at diagnosis, and histological and molecular features [11,13,14,15,18,51,52].

### 2.1. Extent of Resection

The extent of surgical resection has emerged as the predominant predictor of the progression patterns throughout previous population-based cohort studies of PLGG [11,13,14,15,16,17]. Five- and 10-year PFS rates of completely resected PLGGs have been reported to be around 94 and 85%, respectively [11,14]. In incompletely resected PLGGs, progression-free survival was substantially determined by the extent of surgical resection, as 10-year PFS rates of 48% after near-total resection, 18% after partial resection, and 16% after biopsy were reported. Above, a recently published analysis of the pre- and postoperative tumor growth velocity of a cohort of 171 pediatric low-grade gliomas reported a persistent growth deceleration of incompletely resected tumors, while a clear linear negative correlation of percentual extent of resection and postoperative tumor growth velocity was shown [42]. The same study also identified a residual cut-off tumor volume >2.0 cm^3^ associated with a higher risk of radiologically detectable progression post incomplete resection, while the residual tumor mass after initial surgical resection was moreover reported as a risk factor for progression by a further study [23,42]. These data indicate the crucial role of surgical treatment as the mainstay of therapy alongside emerging targeted molecular therapy approaches.

### 2.2. Tumor Localization

The influence of tumor localization on the progression behavior of these tumors has repeatedly been described. Multiple tumor sites or extensive tumor spread at time of diagnosis bearing a significantly higher risk of tumor progression during various disease states have been identified by previous population-based cohort studies. In contrast, supratentorial midline location has been found to be the most prominent tumor site associated with highly progressive disease and disease-related morbidity [11,14,51]. Further tumor locations associated with a higher risk of tumor progression involve the brainstem, spinal cord, and diencephalon [13]. The impact of the tumor location as a prognostic factor, however, may naturally be confounded with the extent of resection of these tumors, as resectability can be severely compromised by surrounding highly eloquent brain tissue, where extensive resection may cause substantial morbidity. In a large multi-institutional analysis involving 798 patients, hypothalamic/chiasmatic tumors demonstrated the most sustained tendency to progress, while a multivariate analysis did not confirm tumor site as an independent risk factor for highly progressive disease, and a strong correlation of tumor site and resection extent was shown [14]. Consistent with these findings, an analysis of the preoperative tumor growth velocity revealed no significant differences comparing tumor growth rates in PLGGs at various tumor locations, while mean postoperative tumor growth rates were highest in PLGGs located in the supratentorial midline, where the lowest mean resection extent was achieved [42]. In contrast, however, a prospective multivariate analysis of two large population-based cohorts involving 1031 patients identified supratentorial midline location as an independent risk factor for highly progressive disease [15]. Comparing the outcome of supra- and infratentorial PLGGs, it should be considered, however, that a reasonable proportion of circumscribed glial and glioneuronal tumors at distinct locations bear additional histone H3 mutations or overexpression of EZHIP, which had not been assessed earlier yet potentially may have influenced the reported outcomes of previously published series [53,54].

### 2.3. Age at Diagnosis

Earlier reports demonstrate a substantial dependence of the clinical progression patterns of PLGGs on the patient’s age at diagnosis. While younger patients are at a higher risk of recurrent treatment progression and inferior treatment outcome, treatment-related sequelae, and tumor-related death, the highest risk of progression has correspondingly been observed in patients < 1 year of age [14,15,18,52,55,56,57,58,59]. Although occasional preselecting treatment studies may point in various directions, age dependency of progression patterns has consistently been reported from major population-based cohort analyses [11,14,15,18,55,56,57,58,59,60,61]. Considering a further distinct predominance of distinct tumor locations among different age groups, various age-dependent progression patterns are currently considered an expression of an evolving tumor microenvironment and probably also some sort of maturation of glial cells across early stages of childhood, promoting differential risks of tumorigenesis and progression of these tumors [11,62]. The underlying mechanistic causes, however, are barely understood, and further investigations are hampered by difficulties in generating representative preclinical models of PLGG, as mentioned above.

### 2.4. Histology

Moreover, previous reports indicate that histology may independently predict progression, as nonpilocytic and diffuse PLGGs have repeatedly been associated with a higher risk of highly progressive disease. Several studies and population-based analyses indicate a significantly higher progression rate in nonpilocytic tumors, diffuse fibrillary histology, and low-grade gliomas assigned to WHO grade 2 [6,11,14,15,58,63,64,65,66]. While distinct tumor location-specific distributions of various histological types of PLGG have been shown, the impact of tumor histology on PFS, however, may be confounded with differences in resectability. It was shown that patients with pilocytic astrocytoma had higher chances of final complete resection [67]. Nonetheless, several large population-based studies confirmed diffuse fibrillary histology as an independent risk factor for a worse PFS in multivariate analyses [11,14,15].

## 3. Aberrant RAS/MAPK Pathway Signaling Drives Tumorigenesis and Tumor Progression in PLGGs

**Figure 2:**
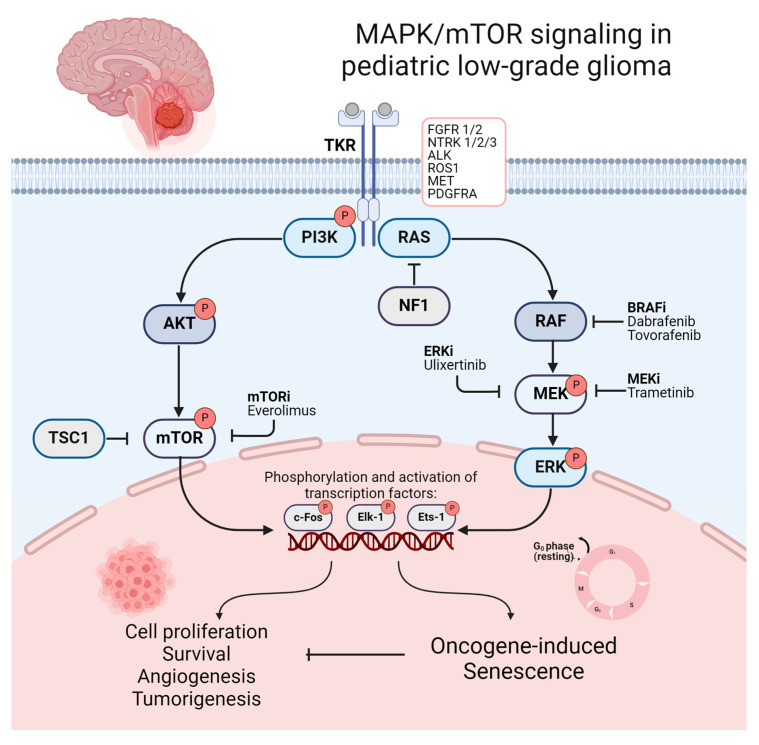
Illustration of MAPK/mTOR signaling and its downstream activation of transcriptional factors resulting in tumorigenesis OIS. Promising molecular therapeutics and their target points in the mTOR/MAPK pathway are additionally illustrated. Created with BioRender.com (accessed on 8 July 2024).

Within the past two decades, in-depth molecular profiling and studies of the genetic landscape of PLGGs identified underlying genetic alterations leading to a frequent activation of the RAS–mitogen-activated protein kinase (RAS/MAPK) pathway as a nearly universal biological feature of these tumors. PLGGs are therefore often referred to as a “single-pathway disease” [68,69,70,71,72,73]. The RAS/MAPK pathway represents one of the best characterized signaling pathways in cell biology. It is involved in regulating essential mechanisms of cell cycle control, cell migration, and angiogenesis, which play a crucial role in tumorigenesis and tumor progression in several malignancies [74,75,76,77]. MAPK/mTOR signaling and its downstream activation of transcriptional factors resulting in tumorigenesis and oncogene-induced senescence (OIS) is illustrated in Figure 2. The first indications of the crucial role of the RAS/MAPK pathway in PLGGs were found in patients with neurofibromatosis type I (NF1). In these patients, germline mutations involving the NF1 tumor suppressor gene cause a loss of function of neurofibromin, a GTPase-activating protein functioning as a negative regulator of RAS [78,79]. This alteration consecutively leads to the development of low-grade gliomas, mainly involving the optic pathway, in up to 20% of cases during childhood [80,81]. Distinct alterations in non-NF1 patients all converging on consecutive RAS/MAPK activation were subsequently discovered. Key mutations driving these tumors include the KIAA1549-BRAF fusion, prevalent in pilocytic astrocytoma (approx. 70%) and rosette-forming glioneural tumors (approx. 30%) in non-NF1 patients, as well as BRAF V600E mutations, commonly found in pleomorphic xanthoastrocytoma (approx. 80%), ganglioglioma (approx. 45%), and pediatric-type diffuse low-grade gliomas (approx. 40%) [69,70,79,82,83,84]. Other less frequent mutations activating the RAS/MAPK pathway include FGFR1/2 alterations, ALK fusions, KRAS mutations, and less frequent mutation or fusions of BRAF with the removal of BRAF’s N-regulatory domain [68,69,79,85,86]. Less common types of PLGGs contain recurrent genetic alterations, including NTRK and the MYB family of transcription factors and fusions involving RAF1 [68,69,87,88]. Protooncogenes of the MYB family are known for their crucial role as transcriptional regulators of proliferation and differentiation, while oncogenic alterations are repeatedly found in PLGGs with diffuse histological features, including angiocentric gliomas [69,79,87,89]. Notably, IDH1/2 mutations, frequently seen in adult gliomas, are rare occurrences in PLGGs and cluster among adolescent patients [90]. Previous observations indicate similar clinical characteristics and progression patterns of IDH1/2-mutant glioma in pediatric and adolescent patients compared to those in adults [90]. Therefore, astrocytoma, IDH-mutant and oligodendroglioma, and IDH-mutant and 1p/19q co-deleted are not covered in this review. An illustration of the frequent and less common genomic alterations of distinct histological types of PLGG is provided in Supplementary Table S1. Beyond contributing to a more profound understanding of tumorigenesis and the driving forces of tumor progression in PLGGs, dissecting the molecular landscape of PLGGs facilitated precise diagnosis and advanced molecular stratification, while also representing a promising avenue for emerging molecular therapies [91].

## 4. OIS as a Potent Tumor-Suppressive Mechanism in RAS/MAPK-Driven Cancers

After first being described in vivo more than 25 years ago, the discovery of a potential induction of cellular senescence by a constitutive activation of oncogenic pathways has shaped an increasingly profound understanding of cellular protective mechanisms against tumorigenesis, beyond potentially providing a promising target for novel combination therapies for various cancers [92,93]. Within the past two decades, similar mechanisms of cell cycle arrest, potentially resulting in growth deceleration and senescence, have been described in several cancers driven by activation of the RAS–RAF–mitogen-activated protein kinase (MAPK) pathway, MYC activation, hyperactivated WNT-β-catenin signaling, activation of the INK4A-RB pathway, or loss of PTEN [93,94,95,96,97,98,99]. In most cases, OIS is characterized by the accumulation of p53 and/or *p16^INK4a^* following the activation of oncogenic pathways, whereas inactivation of p53 or *p16^INK4a^* following additional genetic lesions has been associated with an escape of senescence and malignant transformation of premalignant lesions in several cancers [93,100,101,102,103,104].

OIS in RAS/MAPK-activated neoplasms was among the first to be discovered, and its incremental unravelling has led to an increasing understanding of cellular mechanisms of intrinsic tumor suppression following oncogenic genetic alterations [92,100]. While aberrant RAS/MAPK pathway signaling is attributed a critical role in oncogenesis, cancer cell survival, dissemination, and drug resistance in a variety of human cancers, in vitro studies have repeatedly shown an induction of cell cycle arrest in G_1_ due to the accumulation of cell cycle inhibitors p53 and p16^INK4a^ and a decreased expression of cyclin A following persistent oncogenic RAS/MAPK signaling in murine and human cells [92,100,105,106,107,108,109,110,111]. Previous in vitro analyses implicate a dependency of RAS/MAPK-driven tumorigenesis on additional genetic alterations, resulting in an inactivation of p53 or p16^INK4a^ rendering senescence escape [100,112]. Further investigations of cellular responses to oncogenic RAS signaling in human fibroblasts, however, suggest a correlation of senescence response to the intensity of MAPK activation without dependency on additional genetic lesions, while moderate MAPK activation promoted cell growth, and hyperactivation induced senescence via p16^INK4a^ expression [109].

OIS following MAPK signaling has been associated with various mutations in KRAS and BRAF or upstream growth factor receptors as EGFR and have been translated to multiple cancerogenesis models involving hematologic malignancies, melanoma, and lung cancer [113,114,115,116,117]. Patient-derived cellular PLGG models replicating OIS are therefore useful for further studies in the transcriptional role of the MAPK pathway [31,118].

## 5. Regulation of OIS and in PLGGs: Recent Discoveries and Current Understanding

In pediatric low-grade glial tumors, the pivotal role of OIS has previously been studied in various preclinical MAPK-activated pilocytic astrocytoma models. It was shown that the insertion of a constitutively active BRAF^V600E^ allele into human neural stem cells promoted clonogenic growth, albeit followed by subsequent proliferation arrest and expression of markers of OIS, including acidic β-galactosidase and p16^INK4a^ [119]. Similar observations were reported from BRAF wild-type and mutant astrocytic cell lines characterized by aberrant MAPK signaling, whereas abrogation of OIS could be induced by additional loss of p16^INK4a^ [120].

OIS and the resulting growth arrest due to constitutive upregulation of MAPK signaling has previously moreover been described as the underlying cause of difficulties in the establishment of representative low-grade glioma models, including primary cultures and patient-derived xenograft (PDX) models [119,120,121,122,123]. SV40 large T antigen workflows are usually applied to circumvent OIS in such models [123].

Variable inactivation of OIS, however, enabled the establishment of representative in vitro models for further investigation of OIS in pilocytic astrocytoma. In a patient-derived KIAA1549::BRAF fusion positive pilocytic astrocytoma model, a doxycycline-inducible switching between a proliferative state and OIS by doxycycline-inducible inhibition of TP53/CDKN1A and CDKN2A/RB1 pathways was implemented, while the induction of CDKN1A and an accumulation of TP53 with subsequent G_1_ growth arrest was shown during a senescent state [122,124].

Similar to preclinical models of pilocytic astrocytoma, enhanced expression of hallmarks of senescence including p16^INK4a^ and acidic β-galactosidase, as well as a significantly upregulated mRNA expression of senescence-associated secretory phenotype (SASP) factors, was shown in human pilocytic astrocytoma samples, possibly indicating the significance and translational potential of the observations in preclinical in vitro models to clinical routine [120,122].

Consequently, the indolent growth behavior and versatile progression pattern of pediatric low-grade gliomas has repeatedly been linked with RAS/MAPK-driven OIS in pilocytic astrocytoma by previous authors [122,125,126]. This hypothesis is supported by the observation of Buhl et al. indicating a clear dependency of progression-free survival on SASP factor mRNA expression, which showed a favorable outcome of patients with higher SASP expression in a multi-institutional ICGC PedBrain pilocytic astrocytoma cohort including 110 patients [122]. Other potentially interfering factors, including the extent of resection and postoperative radiotherapy, were excluded in a multivariate analysis.

As the accumulation of p53 or *p16^INK4a^* following the activation of the MAPK pathway plays a paramount role in MAPK-driven OIS, additional CDKN2A alterations are described to facilitate a senescence escape, potentially defining a particularly high-risk, low-grade glioma phenotype [79,119,120]. CDKN2A is known for its endogenous function in G_1_ cell cycle regulation [79,127,128]. The prognostic impact of a concomitant loss of CDKN2A, which is described in a frequency range of 6–20% in PLGGs, has previously been studied in several pediatric low-grade glioma cohorts [79,83,129,130,131]. It was shown that additional homozygous CDKN2A deletion in MAPK-activated BRAF V600E mutant PLGGs contributed independently to poor outcome, therefore constituting a distinct entity with a significantly worse prognosis [83,130,131]. Moreover, the interplay between these cofounding alterations has been shown to foster progression to secondary high-grade glioma (sHGG) in pediatric patients, as a comprehensive molecular analysis of a population-based sHGG cohort showed a significant enrichment of MAPK-activating BRAF mutations and a homozygous loss of CDKN2A in these tumors [132,133]. Notably, as a retrospective longitudinal assessment revealed, these alterations could be traced back to their PLGG counterparts before malignant transformation [132]. Notably, co-occurrence of BRAF V600E mutation and CDKN2A co-deletion moreover showed a significant enrichment in pleomorphic xanthoastrocytoma, a PLGG type known for an increased tendency of malignant transformation to sHGG [69,134,135]. Another example is the recently discovered entity of high-grade glioma with piloid features (HGAP) [136]. Such cases may occur in the setting of NF1, and these tumors frequently harbor co-occurring CDKN2A/B homozygous deletion and/or ATRX stop gain mutations [137]. A significant number of cases show a low-grade precursor lesion originating from the PLGG spectrum [138]. Beyond providing significant aspects for risk stratification, these data moreover support the transferability of the mechanistic model of MAPK-driven PLGGs to clinical practice.

In contrast, additional TP53 mutations appear to play a minor role in the context of OIS escape in the treatment of PLGGs. These DNA repair pathway mutations show a significant accumulation in pediatric high-grade gliomas, particularly in H3K27-altered diffuse midline gliomas, and have shown a notable enrichment in pediatric HGG of the cerebellum in a recently published series [139,140]. Previous data suggest a negative impact of this mutation on the prognosis of pediatric HGGs [141]. However, in PLGGs, TP53 mutations have only been sporadically described, and their detection failed to show a negative prognostic value in a previously published series, indicating that the observation of their crucial impact on OIS escape in preclinical models does not seem to reflect in clinical practice [6,58].

**Figure 3:**
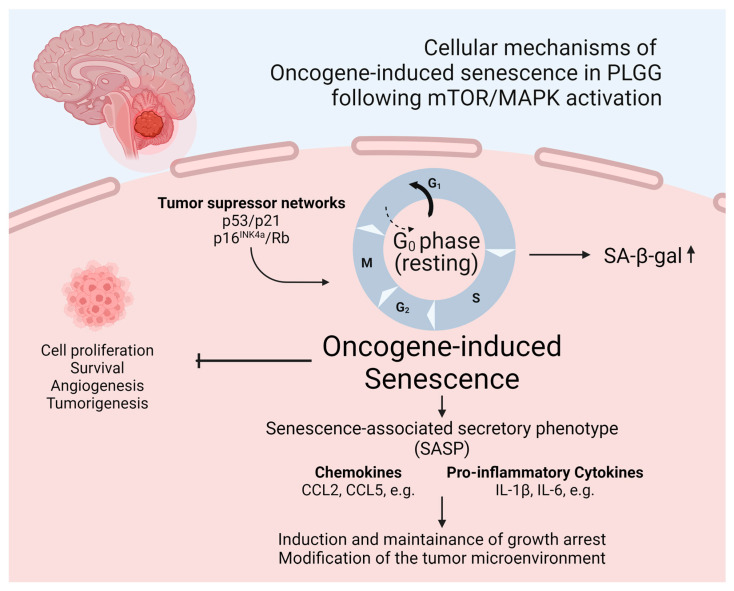
Schematic illustration of OIS fostering the SASP in PLGGs. Created with BioRender.com (accessed on 8 July 2024).

The identification of mediating factors of OIS in pediatric low-grade gliomas was the subject of a previous work by Buhl et al., who analyzed gene and protein expression during a state of proliferation and senescence in a previously mentioned patient-derived KIAA1549::BRAF fusion positive pilocytic astrocytoma (PA) model, which allows for doxycycline-inducible switching between a proliferative state and OIS [122,124]. It was shown that SASP factors, including the pro-inflammatory cytokines IL1B and IL6, were upregulated in pilocytic astrocytoma cells during OIS. This furthermore led to the transcription of SASP factors in an autocrine manner due to downstream activation of NFκB, therefore sustaining the senescent state and resulting a reduced growth of PA cells [122]. Incubation with the anti-inflammatory agent dexamethasone conversely induced regrowth of senescent cells and inhibited the SASP [122]. A schematic illustration of OIS fostering the SASP in PLGGs is provided in Figure 3. These findings may indicate a paramount impact of inflammation on OIS and therefore the growth behavior of these tumors. Considering recently published data of an analysis of pre- and postoperative tumor growth velocity of PLGGs, showing a significant radiologically detectable growth deceleration after subtotal resection, it may be hypothesized that a local inflammatory response to surgical therapy may foster OIS and therefore growth deceleration in residual tumor tissue [42]. Moreover, the in vitro observation of a regrowth of senescent PA cells and suppression of OIS following the incubation with dexamethasone raises the question regarding the safety of the use of anti-inflammatory agents such as dexamethasone in PLGGs, which are frequently applied for symptom control in brain tumor patients with raised intracranial pressure [142]. A retrospective analysis, however, did not show any significant impact of short-term dexamethasone use on tumor growth velocity or PFS in a PLGG cohort [142].

Notably, previous studies of OIS in pediatric low-grade gliomas are confined to preclinical models and tumor samples of pilocytic astrocytoma, while correspondent analyses in distinct histological types of low-grade gliomas are yet missing. Unrestrained translation to distinct histological LGG types should be challenged, paying regard to biological and molecular differences.

## 6. OIS Represents a Promising Target for Senolytic Treatment in PLGGs

The intratumoral heterogeneity with a significantly increased, varying population of senescent cells in pediatric low-grade gliomas may not only serve as an explanation for the indolent growth behavior of these tumors but moreover bears the challenge of potential resistance of senescent compartments to conventional chemotherapy-based treatment regimens [66,125]. In this setting, the potential implementation of senolytic agents may appear as a promising complementary treatment approach.

The evaluation of the therapeutic potential of clearing senescent cells by senolytic drugs through the induction of apoptosis has been the subject of basic research and clinical investigations in a variety of cancers [143,144,145,146,147]. In glial tumors, the potential of Bcl-2 inhibition in overcoming therapy resistance and the clearance of senescent tumor stem cells has previously been demonstrated in vitro and in vivo in glioblastoma [148,149,150,151]. Evidence of increased Bcl-xL levels in pilocytic astrocytoma, indicating a significant fraction of senescent cells and providing a potential target for senolytic BH3-mimetics, underline the potential of this treatment approach in pediatric low-grade gliomas [152]. In a comprehensive in vitro analysis including several patient-derived MAPK-activated pilocytic astrocytoma cell lines, including KIAA1549-BRAF fusions or a BRAF V600E mutation, Selt et al. demonstrated the upregulation of Bcl-xL during a state of cellular senescence, while inhibition of BCL-xL induced mitochondrial apoptosis [152]. These significant findings highlight the potential of BCL-xL inhibition as a promising treatment approach in targeting the senescent compartment in low-grade gliomas, which may be difficult to treat with conventional anti-proliferative agents. While upfront clinical trials are required, limited penetration of the blood–brain barrier, resulting in relatively low plasma:CSF ratios in clinically available BCL-2 inhibitors, provides a challenge that may potentially affect the translation of these promising findings into clinical practice [153].

## 7. The Role of the Tumor Microenvironment in PLGG Tumor Formation and Growth

The crucial role of the tumor microenvironment (TME) as a sophisticated ecosystem in oncogenesis, potential evasion of immune surveillance, and tumor advancement has been studied extensively in a variety of tumor entities and carcinogenesis models within the past decades [154,155,156,157,158,159]. In pediatric CNS tumors—in particular, PLGGs—major questions involving the interaction between the TME and tumor cells, as well the spatial distribution and impact of various infiltrating immune cells on senescence and tumor progression patterns, mostly remain unanswered [160].

The composition of infiltrating immune cells in pediatric gliomas has been the subject of previous analyses using a variety of methods ranging from basic immunohistochemistry to single-cell RNA sequencing. It was shown that primary immune cells found in pediatric gliomas include macrophages, microglia, and T cells, while MAPK-driven low-grade gliomas showed a significantly stronger immune cell infiltration in contrast to their high-grade counterparts, and the composition of infiltrating immune cells showed significant differences to distinct brain tumor entities [160,161,162]. Several previous studies have identified microglia and T cells as the predominant infiltrating cell type in PLGGs, while a significant proportion of resident microglial tumor-associated macrophages (TAMs) has moreover been identified [161,162,163]. More detailed analyses have observed a notably variable extent of infiltrating T cells, with the density of infiltrating T cells being described as highest in ganglioglioma, pleomorphic xanthoastrocytoma, and hypermutated tumors [164]. However, a more recent study describes the detection of a more immunogenic, immune-activating infiltrating cell population in pilocytic astrocytoma compared to ganglioglioma. [165]. The underlying MAPK-activating genetic alteration has been shown to impact the composition of infiltrating immune cells, as a stronger immune infiltration including a higher proportion of pro-inflammatory, activated macrophages and microglia was shown in KIAA1549::BRAF fusion positive compared to BRAF wild-type PLGGs [166,167]. Another study using spatial transcriptomics that has not yet been published found a higher concentration of immune cell-mobilizing chemokines in KIAA1549::BRAF fusion-driven tumors compared to BRAF V600E-mutated tumors [165]. Across various types of pediatric gliomas, previous data suggest a correlation between a strong immune infiltration and significantly prolonged overall survival [168]. Albeit the composition and grade of immune cell infiltration has shown significant variability in pediatric low-grade gliomas, data addressing its prognostic utility and a possible impact on progression patterns are missing [164,169].

While the potential modulation of immunosurveillance by RAS/MAPK-driven OIS in PLGGs has not yet been the subject of detailed investigation, there are clear indications derived from preclinical models of distinct tumor entities that MAPK-driven OIS significantly affects the TME and could thus influence immunological clearance. In a mosaic mouse model of liver carcinoma, it was shown that the secretion of inflammatory cytokines in a senescence state triggered an innate immune response and fostered tumor clearance [170]. In contrast, in a fibroblast model of OIS, it was shown that the expression of inhibitory immune checkpoint molecules such as PD-L1 correlates with gene expression signatures of SASP [171]. In liver tumors, it was shown that senescent tumor cells can alter their surface proteome to evade clearance by the immune system [172]. A previously published work examined the expression of ligands for PD1 (PD-L1 and PD-L2) and CTLA4 (CD80 and CD86), which could potentially influence T-cell-mediated immune clearance, on cells of pilocytic astrocytoma. This study showed a very low expression of these proteins on tumor cells, although a robust expression of MHC class 1 antigens (HLA-A, HLA-B, HLA-C) suggests intact antigen expression. Although there have been no dedicated studies on the modulation of cellular immune response and clearance by the SASP in PLGGs, there are indications that MAPK activation in PLGGs has a crucial impact on immune infiltration. For instance, it has been shown that the likelihood of responding to MAPK inhibitor therapy correlates with the extent of immune infiltration, and rebound growth after MAPK inhibitor withdrawal is associated with the secretion of microglia-recruiting cytokines [39,169]. The potentially crucial interaction of RAS/MAP-driven OIS and immunosurveillance is characterized by many open questions and represents the subject of current research [173].

Remarkably, previous data derived from murine models of PLGGs indicate a strong dependence of tumorigenesis and tumor progression on infiltrating immune cells in these tumors. In a previously published work investigating the impact of BRAF fusion-expressing neural stem cells and microglia in mice, it was shown that increased microglia infiltration induced glioma-like formation of BRAF fusion-expressing neural stem cells, while intercepting chemokine receptor type 2 (CCR2)-mediated microglia recruitment prevented tumor formation [174]. In a genetically engineered neurofibromatosis 1 mouse model, multi-potent low-grade glioma stem cells failed to generate glioma-like lesions in athymic mice lacking T cells or chemotactic receptors such as CCR2 and chemokine c-motif ligand 5 (CCL5), which mediate the interaction of T cells and microglia and moreover were shown to promote low-grade glioma growth [175,176]. It was also shown that various patterns of chemokine expression of both low-grade glioma stem cells and infiltrating immune cells resulted in various growth patterns of these tumors [177,178].

However, both the translation of these findings and further mechanistic studies pursuing a deeper understanding of the impact of the TME on oncogenesis, potential evasion of immune surveillance, and tumor advancement in humanized models of pediatric low-grade gliomas require the development of preclinical in vivo models containing humanized immune systems, as current preclinical models involving xenotransplantation are commonly characterized by impaired immunity [121]. While the multi-faceted mechanisms of interaction between the tumor microenvironment and tumor cells remain mostly unknown, further investigations may crucially impact the future understanding of tumor formation and progression in these tumors.

## 8. Spontaneous Regression and Malignant Transformation in PLGGs: Characteristics of Two Rare Phenomena

Malignant transformation of low-grade gliomas is observed habitually in adult patients, showing a prevalence of up to 72% in previously reported adult LGG cohorts [168,179,180]. In the pediatric demographic, transformation towards high-grade lesions is a rare phenomenon, with reported incidences ranging around 5% [46,47,48,49,50]. This observation moreover underscores the vast biological differences between pediatric and adult LGGs [7]. While the molecular and biological basis has not yet been deciphered, individual factors associated with malignant transformation of PLGGs could be identified. As previously mentioned, a co-occurrence of BRAF V600E mutation and CDKN2A co-deletion has been associated with progression to sHGG, as a significant enrichment in a cohort of sHGG was shown, and these mutations could be traced back to the PLGG counterparts of sHGG bearing these molecular alterations [132]. Notably, co-occurrence of BRAF V600E mutation and CDKN2A co-deletion moreover showed a significant enrichment in pleomorphic xanthoastrocytoma, a PLGG type known for an increased tendency towards malignant transformation [69,134,181,182,183]. Moreover, previous administration of radiotherapy and older age have been suggested as risk factors for a progression towards sHGG, while the latter has shown no statistical significance due to the small sample size in a series of sHGG [16,46,63,184].

Beyond its characteristically indolent growth behavior and a tendency towards growth deceleration and senescence after incomplete resection, occasional spontaneous regression of these tumors is observed in childhood LGGs, representing another distinguishing feature as compared to adult LGGs [7,43,44,45]. Occasional series of incompletely resected cerebellar pilocytic astrocytoma, including long-term follow-up periods of up to 18 years, report high rates of spontaneous regression in 14% to 38% of cases, while solely the extent of surgical resection could be identified as a significant prognostic factor [185,186,187]. Comparable studies including distinct histological types of PLGGs and a comprehensive molecular characterization of tumors showing spontaneous regression are lacking.

## 9. Conclusions and Outlook

Recent in-depth molecular profiling and studies of the genetic landscape of pediatric low-grade gliomas led to the discovery of the paramount role of frequent upregulation of RAS/MAPK and mTOR signaling in the tumorigenesis and progression of PLGGs. The subsequent clinical evaluation of molecular therapies targeting these pathways shows promising results, partly indicating superior response rates compared to established chemotherapy regimens in randomized controlled trials [38]. Rapid implementation into the multimodal treatment approach of these tumors may substantially improve quality of life, particularly in patients suffering from unresectable tumors, multiple progressions, and a high risk of disease-related morbidity and treatment sequelae. A comprehensive understanding of the multilayered clinical and molecular factors determining the natural progression patterns of these tumors may form the foundation for advanced stratification and provide guidance for the management of these tumors within a multimodal treatment approach.

The discovery of the significant role of RAS/MAPK-driven OIS in these tumors may provide an in-depth look into the molecular mechanisms of the indolent growth behavior, versatile progression patterns, and a potential resistance of senescent compartments to conventional chemotherapy-based treatment regimens. In this setting, the potential implementation of senolytic agents may appear as a promising complementary treatment approach. A recently published comprehensive analysis provides proof of principle for the induction of apoptosis in senescent pilocytic astrocytoma cells by Bcl-xL inhibition [152].

The incremental discovery of the multi-layered cellular mechanisms, including molecular alterations promoting aberrant RAS/MAPK and mTOR signaling, reversible complexly regulated OIS, and a bidirectional dependence of these tumors on their tumor microenvironment, will shape an integrated mechanistic model of the underlying biology of these tumors. This model needs to be incorporated into in vivo and in vitro models for preclinical drug development and future studies of the biology of PLGGs.

## Supplementary Materials

The following supporting information can be downloaded at: https://www.mdpi.com/article/10.3390/cells13141215/s1, Table S1: Illustration of the frequent and less common genomic alterations of distinct histological types of pLGG, as well as the state-of-the-art treatment based on the current European standard clinical practice recommendations for primary pediatric low-grade gliomas. Reference [188] is cited in Supplementary Materials.
