# Supplementary material for: Dissecting the Natural Patterns of Progression and Senescence in Pediatric Low-Grade Glioma: From Cellular Mechanisms to Clinical Implications
Source: Cells. 2024 Jul 19;13(14):1215. doi: 10.3390/cells13141215 (PMC11274692; doi:10.3390/cells13141215)
Supplement: Supplementary file 1 [file cells-13-01215-s001.zip › cells-3060557-supplementary.pdf]

Histologic types	Frequent genomic alterations	Less common alterations
**Circumscribed astrocytic glioma**		
Pilocytic astrocytoma	KIAA1549::BRAF (~70%)	RAF fusions; BRAF, FGFR1 or KRAS SMVs; NF1
Pleomorphic xanthoastrocytoma	BRAF V600E mutation (~90%), CDKN2A codeletion	BRAF mutations; RAF1, NF1 and NTRK1/2 fusions
Subependymal giant cell astrocytoma	TSC1/2 biallelic inactivation (~90%)	BRAF V600E mutation
**Pediatric-type diffuse low-grade gliomas**		
Diffuse astrocytoma, MYB- or MYBL1-altered	MYB- or MYBL fusions	
Angiocentric glioma	MYB alterations (~80%)	
Polymorphous low-grade neuroepithelial tumor of the young	FGFR1 tandem duplication; FGFR1 or BRAF SNVs	BRAF mutations; NTRK2 duplication
Diffuse low-grade glioma, MAPK pathway altered	FGFR2/3 fusions (~40%), BRAF V600E (~40%)	NTRK1-3 fusions, MAP2K1 or FGFR2 mutations
**Glioneuronal and neuronal tumors**		
Ganglioglioma	BRAF V600E mutation (~50%)	KRAS, FGFR1 or NF1 SNVs; RAF1 or ABL2 fusions
Diffuse leptomeningeal glioneuronal tumor (DLGNT)	1p or 1p/19q co-deletion + BRAF fusion	RAF1 or NTRK1-3 fusions; BRAF or RAF1 SNVs
Rosette-forming glioneuronal tumor	PIC3CA or FGFR1 mutation, KIAA1549::BRAF	
Desembryoblastic neuroepithelial tumor	FGFR1 tandem duplication, FGFR1 SNV, FGFR1 fusion	BRAF V600E mutation

State-of-the-art treatment

Interdisciplinary, individualized approach considering age, tumor location, underlying cancer predisposition syndromes, tumor progression and neurological symptoms

**Surgery**		
Surgical resection	Supported by microsurgery, intraoperative imaging (iUS, iMRI), neuromonitoring	Surgical resection is the mainstay of therapy.
Biopsy	If feasible via endoscopic surgery or supported by stereotaxy	If not feasible, biopsy is recommended excepting NF1-related optic pathway glioma.
**Chemotherapy**		
Carboplatin + vincristine	Induction phase (24 weeks) + Consolidation therapy (61 weeks)	First-line non-surgical therapy in unresectable pLGG in cases of tumor progression, neurologic symptoms or infants below 12 months with chiasmatic-hypothalamic tumors
Vinblastine monotherapy	Weekly application, 70 weeks	
**Targeted therapy**		
Dabrafenib plus trametinib	First-line non-surgical treatment in patients ≥1 year of age and BRAF V600E mutation	Unknown issues include optimal treatment duration and potential very late adverse effects
Tovorafenib	LGG with RAF alteration relapsing after first-line therapy	
Everolimus	First-line non-surgical treatment in patients with Supependymal giant cell astrocytoma	
**Radiotherapy**		
Although the role of radiotherapy has declined in recent years, conventional radiation or proton therapy with recommended doses between 50.4 and 54 gray remains an option particularly as a second-line treatment in selected cases after careful consideration and multidisciplinary agreement. Potential cases include inoperable, recurrent tumors with limited or no response to systemic therapy, a high risk of significant morbidity due to tumor progression and a tolerable risk of radiotherapy-related neurocognitive effects.		

Table S1 Illustration of the frequent and less common genomic alterations of distinct histological types of pLGG, as well as the state-of-the-art treatment based on the current European standard clinical practice recommendations for primary pediatric low-grade gliomas [189].
